# Limited efficacy of adalimumab in the acute phase of serpiginous choroiditis refractory to corticosteroid and cyclosporine, a case report

**DOI:** 10.1186/s12886-019-1104-3

**Published:** 2019-04-24

**Authors:** Kazunori Noda, Akio Oishi, Akihito Uji, Saori Tanaka, Akitaka Tsujikawa

**Affiliations:** Department of Ophthalmology and Visual Sciences, Kyoto University Graduate School of Medicine, 54 Shogoin Kawahara-cho, Sakyo-ku, Kyoto, 606-8507 Japan

**Keywords:** Serpiginous choroiditis, Chorioretinal degeneration, Geographic choroiditis, Geographic choroidopathy, Posterior uveitis, Relentless placoid chorioretinitis, Serpiginous choroidopathy

## Abstract

**Background:**

The optimal treatment of serpiginous choroiditis is not established. While recent reports indicate the efficacy of adalimumab, there is limited evidence. We present a case of serpiginous choroiditis refractory to steroids, immunosuppressants, and adalimumab.

**Case presentation:**

An 18-year-old woman presented with severe vision loss in both eyes. A fundus examination revealed a foveal grayish-white lesion, and optical coherence tomography revealed outer retinal damage. She was diagnosed with serpiginous choroiditis and treated with steroid pulse therapy, but the disease progressed continuously. The addition of sub-Tenon’s injection of triamcinolone and oral cyclosporine did not change the disease course. We also administered subcutaneous injections of adalimumab, but even with the intensive treatment, the retinal lesions and subsequent atrophy progressed. Her right and left visual acuity declined from 20/22 to 20/66 and 20/200, respectively, during the 9 months of follow-up.

**Conclusion:**

Here, we report a case of serpiginous choroiditis refractory to corticosteroids, immunosuppressants, and adalimumab. Further studies are needed to establish the optimal treatment for such cases.

## Background

Serpiginous choroiditis was first described in 1932 by Junius as “peripapillary retinochoroiditis,” after which there were various designations and reports on the subtypes [1]. The disease is characterized by bilateral, chronic, progressive, and recurrent inflammation of the choroid, choriocapillaris, and retinal pigment epithelium [1, 2]. The disease is prevalent in healthy, young to middle-aged adults irrespective of race [1]. Infectious diseases such as tuberculosis may present serpiginous-like choroiditis and must be ruled out. The optimal treatment is yet to be established because of the rarity and unknown etiology of the disease. Corticosteroids and immunosuppressants, such as cyclosporine and azathioprine, are commonly prescribed. Recently, some reports showed the efficacy of biological drugs such as infliximab [3] and adalimumab [4, 5] and intravenous pulse cyclophosphamide therapy [6]. However, these are single case reports, so the efficacy of the drugs may have been overestimated. In this paper, we present a case of serpiginous choroiditis refractory to adalimumab, corticosteroids, and cyclosporine.

## Case presentation

An 18-year-old woman was referred to our hospital with the complaint of central visual field defect in the right eye for 1 week and in the left eye for 3 days.

Her medical history was unremarkable except for pediatric asthma and appendicitis. She reported no raw meat consumption and had a dog until a year ago. She received vaccination for human papillomavirus 3 years ago. The best corrected visual acuity was 20/22 in both eyes.

**Fig. 1:**
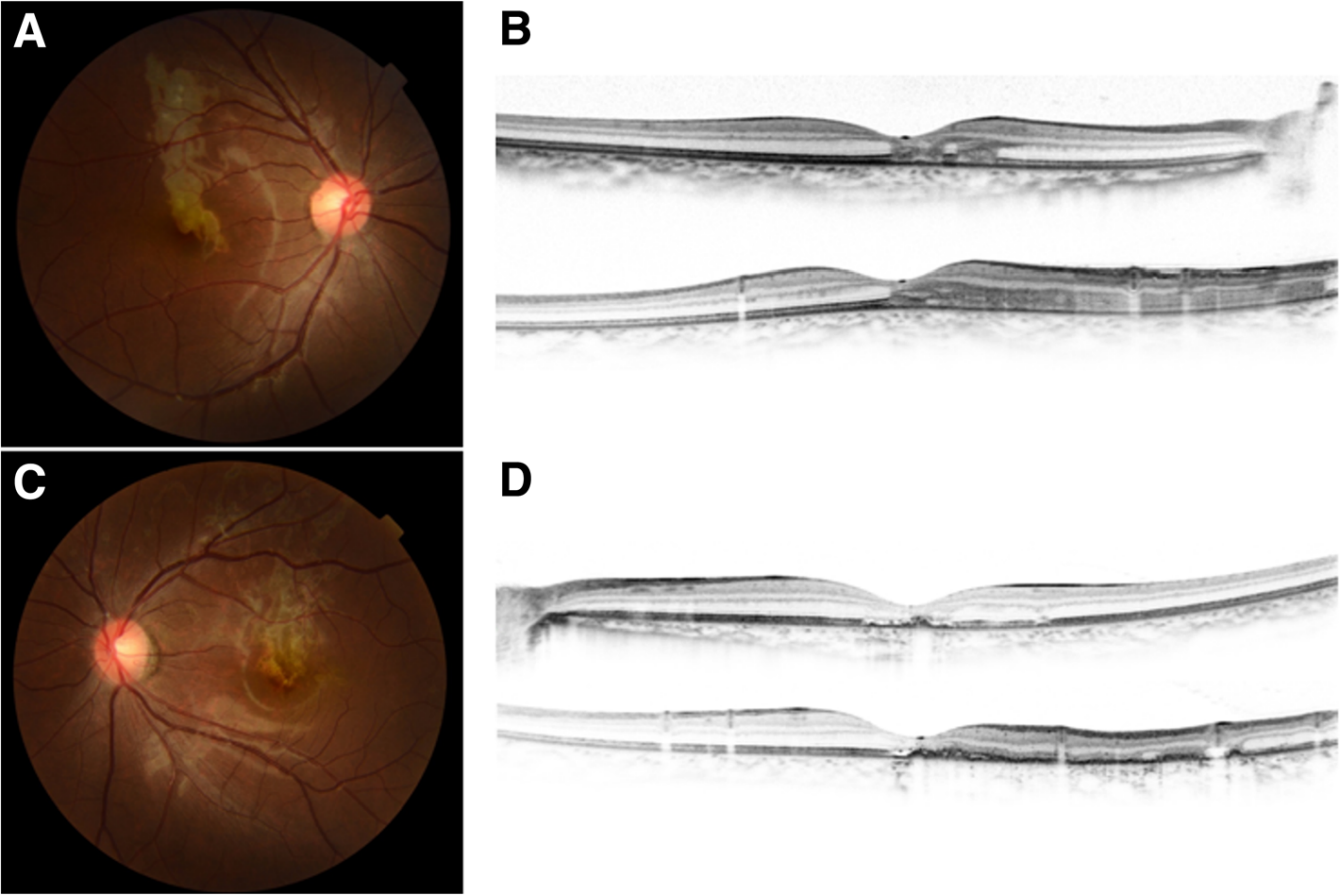
Fundus photograph (**a** and **c**) and optical coherence tomography (OCT) images (**b** and **d**) of an 18-year-old patient with serpiginous choroiditis. A grayish-white exudative lesion is observed in the macula area. The corresponding OCT image shows a hyperreflective appearance in the outer retina

**Fig. 2:**
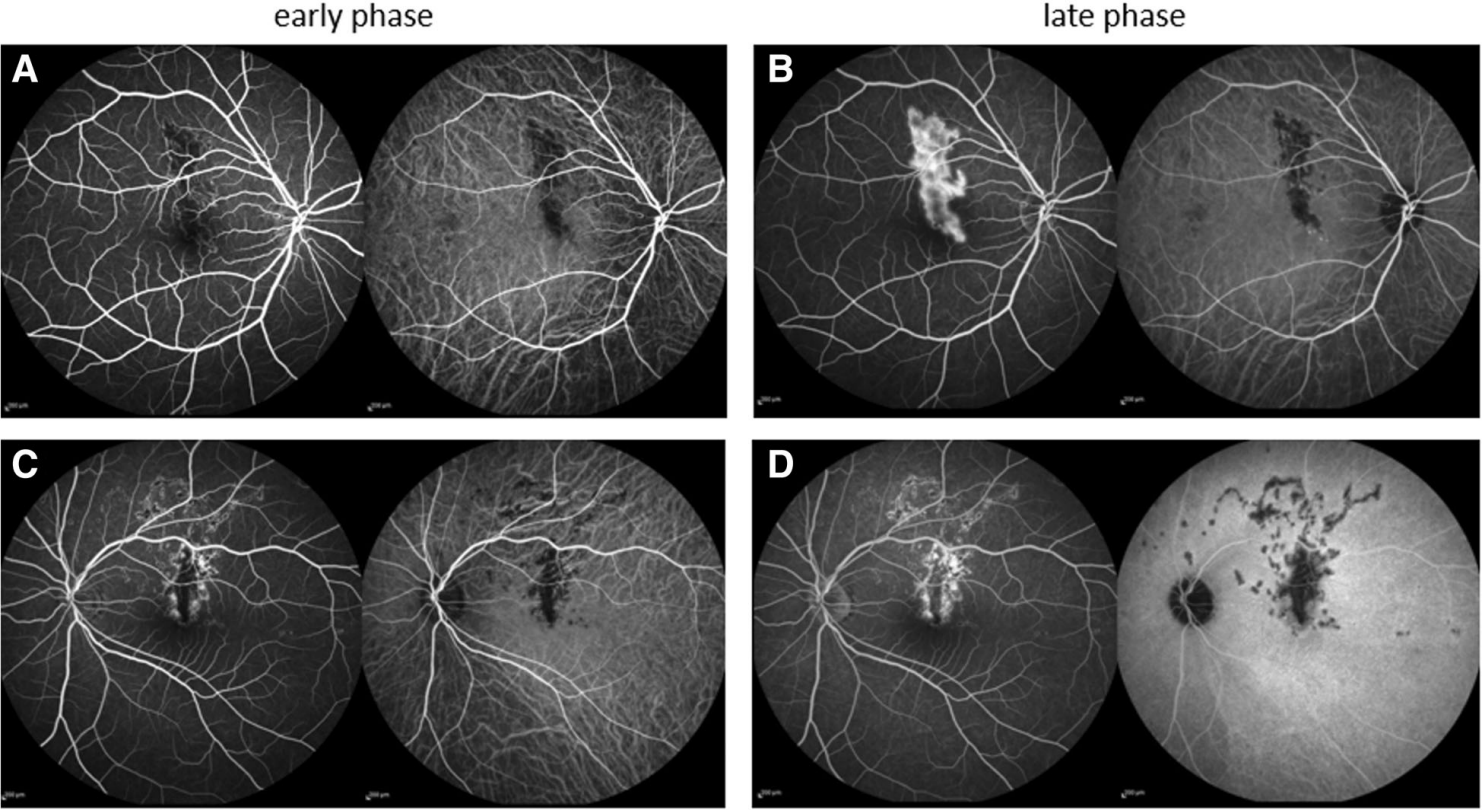
Fluorescein and indocyanine angiography (FA and ICGA in the left and right panels, respectively) images of the patient. On FA, the lesion appeared hypofluorescent in the early phase (**a** and **c**) and turned hyperfluorescent in the late phase (**b** and **d**). Meanwhile, ICGA showed a hypofluorescent appearance in both the phases

The anterior segment examination was unremarkable in both eyes. The fundus examination showed bilateral grayish-white retinal lesions around the macula, and the optical coherence tomography showed corresponding hyperreflectivity and thinning of the outer retina (Fig. 1**)**. The features of tuberculous serpiginous-like choroiditis such as vitreous hyper-reflective spots, intraretinal edema, sub-retinal pigment epithelium drusenoid deposits, and choroidal granulomas were not present [7]. The lesion was hypofluorescent and hyperfluorescent in the early and late phases, respectively, on fluorescein fundus angiography (FA). The lesion was hypofluorescent from the early to late phase on indocyanine green angiography (ICGA) (Fig. 2).

The intraocular pressures were 18 and 15 mmHg in the right and left eyes, respectively.

No abnormalities were detected with blood tests except for a mild increase of C-reactive protein (0.4 mg/dL) and white blood cells (10,020/μL). We performed QuantiFERON tests at the initial presentation and 2 weeks later, which showed negative results.

She was diagnosed with serpiginous choroiditis and treated with prednisone 40 mg/day.

**Fig. 3:**
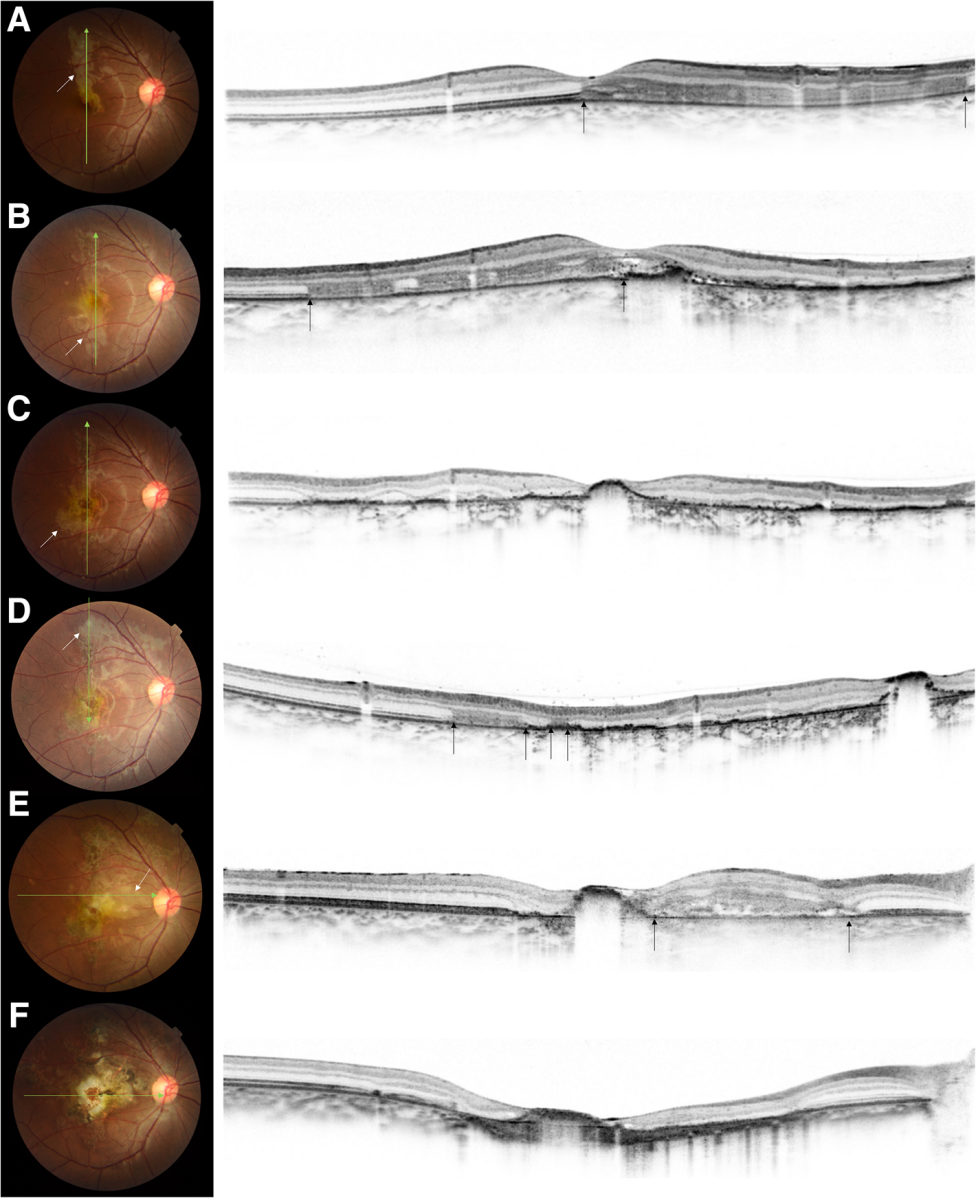
Fundus photographs and optical coherence tomography (OCT) images of a serpiginous choroiditis patient. **a** At initial presentation. **b** The lesion enlarged despite 6 days of oral prednisone (white arrow). The corresponding OCT revealed a hyperreflective lesion in the outer retina (black arrows). **c** Day 16. Even with steroid pulse therapy and sub-Tenon’s injection of triamcinolone, the lesion had continued to enlarge (white arrow). The original lesion rapidly progressed to an atrophy. **d** Day 24. We started cyclosporine, but the new lesion appeared upper to the optic disc (white arrow). The OCT showed development of the hyperreflective lesion (black arrows). **e** Day 41. Two weeks after the adalimumab injection., the lesion had enlarged to reach the nose (white arrow, black arrows). **f** Month 9. The disease had progressed for 8 months and left a fibrotic scar in the macula. No new lesions were observed after the 9th month

On the 6th day, the retinal lesion was enlarged, and the outer retinal damage had progressed (Fig. 3b). Thus, transvenous methylprednisolone (mPSL; 1 g/day) was administered for 3 days.

After the initiation of the steroid pulse therapy, the subjective symptoms improved. However, the grayish-white retinal lesions continued to enlarge in both eyes. The disease progression was not controlled despite 3 more days of mPSL (1 g/day), following 60 mg of oral prednisone. Sub-Tenon’s triamcinolone acetonide injection (20 mg) was administered to the left eye and then to the right eye. (Fig. 3c).

We added oral cyclosporine (300 mg), and prednisone was switched to betamethasone (7 mg). The trough blood cyclosporin concentration was monitored and controlled at around 200 ng/mL. While the initially affected areas changed to atrophic scars, new lesions appeared adjacent to or away from the initial lesion (Fig. 3d). Subcutaneous injections of adalimumab (80 mg) were started on the 27th day, and intravitreal injections of triamcinolone acetonide (20 mg) were administered in the right eye on the 34th day, but they could not stop the disease progression. Oral cyclosporine (300 mg) and betamethasone (3 mg) and biweekly subcutaneous injections of adalimumab (40 mg) were continued, but the retinal lesions progressed up to 8 months (Fig. 3e). Her visual acuity declined to 20/66 and 20/200 in the right and left eyes, respectively. Both eyes showed a similar course during the observation period. The disease progressed up to the 9th month, but no new lesions were observed thereafter (Fig. 3f**)**.

## Discussion and conclusions

We presented a refractory case of serpiginous choroiditis.

The disease progressed for 9 months despite the use of adalimumab. The course was inconsistent with recent two case reports showing the efficacy of adalimumab in serpiginous choroiditis refractory to conventional treatment [3, 4]. Both studies reported favorable responses, 1 month after the adalimumab treatment [3, 4]. However, adalimumab was used in the chronic phase in these reports (about 3 and 8 years after the onset, respectively). The progression was controlled with adalimumab after 8 months in the present case. While we cannot draw definite conclusions from a single case, the effect of adalimumab in the acute phase of refractory serpiginous choroiditis may not be as good as was previously reported for the chronic phase. Further studies are needed to establish the optimal treatment.

The present case indicated the utility of optical coherence tomography (OCT) to monitor.

the disease progression. The lesion appeared as a hyperreflective area in the outer retina and progressed to retinal atrophy. The grayish-white lesion could also be confirmed in fundus examinations, but differentiating between the fibrotic scar and the recurred lesion was sometimes challenging (Fig. 3f). The OCT-based treatment strategy, as established in age-related macular degeneration, needs to be tested in future studies.

Differentiating between serpiginous choroiditis and tuberculous serpiginous-like choroiditis is not always easy. In the present case, the failure of response to immunosuppressive treatments made us re-consider tuberculous serpiginous-like choroiditis and other infectious conditions. Additionally, the multifocal appearance was suggestive of tuberculous serpiginous-like choroiditis [8]. However, the repeated QuantiFERON test results were negative, and the OCT features reported in tuberculous serpiginous-like choroiditis were absent in the present case. Blood tests did not indicate other possible conditions such as syphilis or herpes. Considering the side effects of anti-tuberculous and anti-viral treatments, we continued immunosuppression and succeeded to cease the disease progression. A careful evaluation is necessary in such refractory cases.

## Abbreviations

FA: Fluorescein angiography
ICGA: indocyanine green angiography
mPSL: methylprednisolone
OCT: optical coherence tomography
